# The Strawberry Pathogenesis-related 10 (PR-10) Fra a Proteins Control Flavonoid Biosynthesis by Binding to Metabolic Intermediates*

**DOI:** 10.1074/jbc.M113.501528

**Published:** 2013-10-16

**Authors:** Ana Casañal, Ulrich Zander, Cristina Muñoz, Florine Dupeux, Irene Luque, Miguel Angel Botella, Wilfried Schwab, Victoriano Valpuesta, José A. Marquez

**Affiliations:** From the ‡ Instituto de Hortofruticultura Subtropical y Mediterránea (IHSM-UMA-Consejo Superior de Investigaciones Científicas), Departamento de Biología Molecular y Bioquímica, Universidad de Málaga, 29071 Málaga, Spain,; the § European Molecular Biology Laboratory, Grenoble Outstation, 6 rue Jules Horowitz, 38042 Grenoble, France,; the ¶ Unit of Virus Host-Cell Interactions, Université Grenoble Alpes-EMBL-CNRS, 6 rue Jules Horowitz, 38042 Grenoble, France,; the ‖ Department of Physical Chemistry and Institute of Biotechnology, University of Granada, Campus Fuentenueva s/n, 18071 Granada, Spain, and; ** Biotechnology of Natural Products, Technische Universität München, 85354 Freising, Germany

**Keywords:** Crystal Structure, Flavonoids, Plant Biochemistry, Plant Molecular Biology, Secondary Metabolism

## Abstract

Pathogenesis-related 10 (PR-10) proteins are involved in many aspects of plant biology but their molecular function is still unclear. They are related by sequence and structural homology to mammalian lipid transport and plant abscisic acid receptor proteins and are predicted to have cavities for ligand binding. Recently, three new members of the PR-10 family, the Fra a proteins, have been identified in strawberry, where they are required for the activity of the flavonoid biosynthesis pathway, which is essential for the development of color and flavor in fruits. Here, we show that Fra a proteins bind natural flavonoids with different selectivity and affinities in the low μm range. The structural analysis of Fra a 1 E and a Fra a 3-catechin complex indicates that loops L3, L5, and L7 surrounding the ligand-binding cavity show significant flexibility in the apo forms but close over the ligand in the Fra a 3-catechin complex. Our findings provide mechanistic insight on the function of Fra a proteins and suggest that PR-10 proteins, which are widespread in plants, may play a role in the control of secondary metabolic pathways by binding to metabolic intermediates.

## Teaser

**Background:** Suppression of Fra a gene expression leads to down-regulation of color-producing flavonoid biosynthesis in strawberry.

**Results:** Fra proteins can bind natural flavonoids, which induce conformational changes in conserved loop regions.

**Conclusion:** Fra a proteins control flavonoid biosynthesis through binding to metabolic intermediates.

**Significance:** PR-10 proteins may play a role in the control of secondary metabolism through binding of metabolites to their ligand-binding cavities.

## Introduction

The family pathogenesis-related 10 (PR-10)^5^ proteins comprises a large number of sequences widely distributed among seed plants (1). However, their function is still poorly understood. PR-10 proteins were initially characterized by their increased expression levels in response to infection by plant pathogens and under abiotic stress conditions. Today, a large number of PR-10 genes have been identified in different species, showing a diversity of expression patterns under both normal growth and stress conditions (1, 2). Some PR-10 proteins such as the white birch Bet v 1 and the apple Mal d 1 are highly abundant in pollen and fruits, respectively, and are responsible for allergic reactions, including seasonal and food allergies (3–8). PR-10 proteins belong to the START superfamily. These proteins adopt a helix-grip fold with an internal cavity capable of binding hydrophobic ligands (2, 9–11). Only two protein families within the START superfamily have been extensively characterized at a functional and structural level: the lipid transport proteins, which are involved in non vesicular transport of lipids in eukaryotic cells (10, 12) and the plant PYR/PYL/RCAR proteins that function as intracellular receptors for the plant hormone abscisic acid (13–18). Some PR-10 proteins, including Bet v 1, the mung bean cytokinin-specific binding protein and the *Prunus* LPR10 protein, have been found to bind to a series of artificial and natural hydrophobic molecules, including cytokinins and phytosteroids (2, 19–25). However, the functional relevance of these interactions remains unclear. Recently, three new members of the PR-10 family, Fra a 1E, Fra a 2, and Fra a 3, have been identified and shown to play an important role in the control of phenylpropanoids and flavonoids biosynthesis in strawberry fruits (26–28).

**FIGURE 1.**
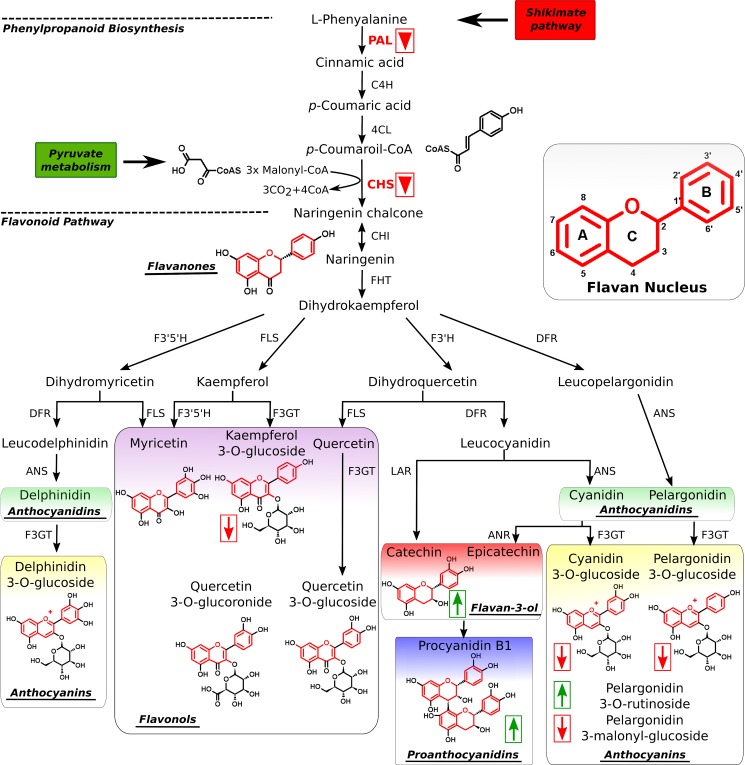
**The phenolic compound biosynthesis pathway.** A schematic representation of the phenylpropanoid and flavonoid biosynthesis pathway is shown. Major families of flavonoid compounds are highlighted. Flavonoids are characterized by the presence of the flavan nucleus with A, B, and C rings as indicated (*inset*). Final products of the flavonoid pathway such as pelargonidin 3-*O*-glucoside, are often glycosylated at the position 3 of the C ring of the flavan nucleus. Suppression of Fra a protein expression affects the expression of phenylalanine ammonia lyase (*PAL*) and chalcone synthase (*CHS*) genes (*red inverted triangles*) and alters phenolic compound accumulation with an increase in the levels of catechin and a decreased accumulation of anthocyanins (as indicated by *arrows*) (28).

Flavonoids and phenolic compounds are among the most important secondary metabolites in plants. In addition to color and flavor development, they participate in many aspects of plant biology, including UV protection, as antioxidants, auxin transport regulators, and defense compounds against pathogens (29–33). Thus, injury by pathogens or pests induces the accumulation of flavonoids and other phenolic compounds with antimicrobial activity (34). Flavonoids are also exuded by plant roots and act as signals that modify the transcriptional activity of nodulation genes in nitrogen-fixing bacteria, thereby promoting symbiotic association (35, 36). Other flavonoids have been implicated in pollen germination, seed resistance to pests and numerous other processes (36, 37). The effect of dietary flavonoids in human health is also a subject of study due to their antioxidative and anticarcinogenic activities (38). Flavonoids are synthesized via the phenylpropanoid and flavonoid pathways (see Fig. 1) (30, 32). The first step in the phenylpropanoid pathway is catalyzed by the enzyme phenylalanine ammonia-lyase (PAL) and leads to the production of cinnamic acid from l-phenylalanine. PAL is the gateway enzyme to the synthesis of phenolic and flavonoid compounds as well as many other secondary metabolites (32). In *Arabidopsis* and other species, *PAL* gene expression is responsive to developmental and environmental clues such as wounding, pathogen infection, or UV radiation, among others (39–43). Another important step in the synthesis of flavonoids is the production of naringenin, which is the first product in the pathway with a flavan structure and from which many other flavonoids are derived (see Fig. 1). This step is catalyzed by the enzyme chalcone synthase. Many of the final products of the flavonoid biosynthesis pathway accumulate as *O*-glycosyl derivatives at the position 3 of the C ring of the flavan nucleus and are accumulated in the vacuole or secreted through the plasma membrane into the apoplastic space (44). A number of flavonoids that account for color of the fruit and contribute significantly to its taste are produced in the strawberry fruit in a developmental-specific pattern (29, 45). Proanthocyanidins (condensed tannins) are mostly produced in the young fruits that make them bitter, whereas anthocyanins, mostly pelargonidin-3-*O*-glucoside and cyanidin-3-*O*-glucoside (see Fig. 1), which confer color, are abundant in the later stages of fruit maturation.

The strawberry Fra a transcripts are present in most plant organs; however, they show maximal expression levels in open flowers, fruits, and roots, depending on the member of the family (28). The first evidence of the involvement of Fra a proteins in the control of the synthesis of flavonoids was provided by Emanuelsson and co-workers (27), who reported that fruits of colorless cultivars showed very low levels of Fra a 1 protein expression in contrast to red-colored fruits. Later, *Fra a RNAi* silencing experiments showed that suppression of the expression of Fra a proteins in strawberry fruits led to decreased accumulation of the main flavonoids responsible for the red color of fruits, including cyanidin 3-*O*-glucoside and pelargonidin 3-*O*-glucoside (see Fig. 1), whereas other aspects of fruit maturation were unaffected (28). Other flavonoids such as kaempferol 3-*O*-glucoside and pelargonidin 3-malonyl-glucoside also showed decreased levels in silenced fruits, whereas other intermediate metabolites of the flavonoid pathway such as catechin and proanthocyanidins accumulated at higher levels (see Fig. 1). Interestingly, silencing of Fra a proteins in strawberry also produced a decrease in the expression levels of genes encoding for PAL and chalcone synthase in the fruits, showing that Fra a proteins are required for the expression of structural genes in the flavonoid biosynthesis pathway and suggesting that their function may be regulatory (28). These studies indicated that the Fra a proteins participate in the control of flavonoid biosynthesis in strawberry fruits. Recently, a solution structure of the Fra a1 protein has been described (46). However, the molecular basis for the function of the Fra proteins remains unknown.

In this work, we show that strawberry Fra a proteins bind natural flavonoids, providing a basis for their function in the control of flavonoid metabolisms. Moreover, we present crystallographic structures of Fra a 1E and Fra a 3 in complex with catechin. The analysis of these structures shows that flavonoid binding is associated with conformational changes in critical loop regions providing for the first time a molecular basis for the function of Fra a proteins in the control of flavonoid biosynthesis.

## EXPERIMENTAL PROCEDURES

### 

#### 

##### Cloning Expression and Purification

Isolation of *Fra a 1E*, *Fra a 2,* and *Fra a 3* cDNAs ORFs from strawberry has been described previously (28). *Fra a 1E*, *Fra a 2,* and *Fra a 3* ORFs were PCR-amplified and cloned into pETM11 (47) to produce expression constructs F1-pETM11, F2-pETM11, and F3-pETM11, respectively. These constructs include an N-terminal His_6_ tag followed by the TEV cleavage sequence. After TEV cleavage, only three amino acids (Ala-Met-Ala) remain at the N-terminal end of the proteins. Purification of Fra a proteins was carried out as described by Casañal *et al.* (48). Briefly, *Escherichia coli* BL21(DE3) cells were transformed with either of the Fra a constructs and grown in 2 liters of LB medium containing 50 μg/ml kanamycin to an OD at 600 nm of 0.6–0.8. At this point, 1 mm isopropyl 1-thio-β-d-galactopyranoside was added, and the cells were harvested after overnight induction at 20 °C and stored at −80 °C before purification. The cells were resuspended in 180 ml of lysis buffer (30 mm Tris, pH 7.5, 500 mm NaCl, 15 mm imidazole, 1 mm β-mercaptoethanol, and protease mixture inhibitor) and lysed with a microfluidizer (Microfluidics). A cleared lysate was obtained after centrifugation at 20,000 rpm for 45 min. The protein extract was incubated in 25 ml of nickel-nitrilotriacetic acid agarose and washed with 125 ml of lysis buffer. The bound protein was eluted with a buffer containing 30 mm Tris, pH 7.5, 300 mm NaCl, 250 mm imidazole, and 1 mm β-mercaptoethanol. The His_6_ tag of the purified proteins was removed by digestion with the His_6_-tagged version of the TEV protease. The cleaved samples were incubated with nickel-nitrilotriacetic acid to remove the undigested proteins the TEV protease and other contaminants. The correct size of the recombinant proteins was verified by SDS-PAGE. Purified Fra a proteins were extensively dialyzed against sample buffer (30 mm Tris-HCl, pH 7.5, 150 mm NaCl, and 1 mm β-mercaptoethanol), concentrated to 60 mg/ml, and flash-frozen in liquid nitrogen for storage at −80 °C.

##### Isothermal Titration Calorimetry (ITC)

ITC experiments were performed using an ITC200 micro-calorimeter (MicroCal, Inc.). Prior to the experiments protein solutions were dialyzed against sample buffer (20 mm HEPES, pH 7.5, 1 mm β-mercaptoethanol). Ligands were dissolved in the dialysis buffer, and the pH was carefully adjusted to pH 7.5. All solutions were filtered and degassed. ITC measurements were performed with the three Fra a isoforms and the following flavonoid compounds: (+)-catechin, quercetin-3-*O*-glucuronide, myricetin, and pelargonidin-3-*O*-glucoside. A number of other phenolic compounds were tested with individual Fra a isoforms according to their specific spatial and developmental expression profiles during fruit ripening (28, 45, 49, 50). This included quercetin-sophoroside with Fra a 1E, cyanidin-3-*O*-glucoside and naringenin with Fra a 2 and procyanidin-B2 and quercetin-3-*O*-glucoside with Fra a 1E and Fra a 3, respectively. Early precursors of the phenylpropanoid pathway (l-phenylalanine, coumaric acid, cinnamic acid, and caffeic acid) were tested with Fra a 2. Thermograms were recorded at 25 °C and typically involved 26 injections of 1.5 μl, with 3-s intervals between injections with flavonoid compounds as injectants and proteins loaded in the calorimeter cell. Initial protein concentrations varied from 20 to 100 μm, whereas injectant concentrations were between 0.4 and 3 mm. Higher injectant concentrations were not possible due to limited solubility of the ligands in aqueous solutions. The heat associated with the binding process was calculated as the difference between the heat of reaction and the corresponding heat of dilution, as obtained from independent titrations of the ligand into buffer. The resulting binding isotherms were analyzed and fitted through non-linear least-squares to an appropriate thermodynamic model with the MicroCal Origin software (MicroCal, Inc.).

##### Crystallization X-ray Data Collection and Structure Solution

Fra a 1E, Fra a 2, and Fra a 3 proteins were assayed for crystallization by the vapor diffusion method, the High Throughput Crystallization Laboratory of the EMBL Grenoble Outstation (51) in the presence and absence of the flavonoid compounds indicated in the previous section. The Fra a 1E protein produced crystals in two different crystal forms. Crystal form A was obtained at a protein concentration of 26 mg/ml and using 0.2 m ammonium sulfate, 0.1 m sodium cacodylate, pH 6.5, 15% PEG 8000 as precipitant, and at an incubation temperature of 20 °C. Crystal form B was obtained by diluting purified Fra a 1E to a concentration of 26 mg/ml in sample buffer and adding solid (+)-catechin powder in excess. This solution was incubated at 4 °C overnight in an overhead shaker and centrifuged at 14,000 × *g* in a bench top centrifuge. The supernatant was used for crystallization. Crystals were obtained in 0.1 m trisodium citrate dihydrate, 0.1 m Tris hydrochloride, pH 8.5, and 5% PEG 400. The Fra a 3 protein produced crystals only in the presence of (+)-catechin. The best diffracting crystals were obtained by adding (+)-catechin powder in excess to a 26 mg/ml Fra a 3 solution as described for Fra a 1E crystal form B. Fra a 3-catechin crystals were optimized by the vapor diffusion method by mixing 1 μl of protein and 1 μl of precipitant solution (2.25 m sodium malonate, pH 7.0) equilibrating against a reservoir containing 0.5 ml of precipitant solution. Crystals in the shape of flat hexagonal prisms appeared within 2 days. Crystals were flash-frozen in liquid nitrogen using 15% glycerol as cryoprotectant. The Fra a 2 protein did not produce any crystals, neither alone nor in the presence of any of the mentioned flavonoids. X-ray diffraction data were collected at the ID14-4, ID23-2, and ID14-1 beamlines of the ESRF for Fra a 1E crystal form A, Fra a 3-catechin and Fra a 1 E crystal form B, respectively. Crystallographic data reduction and scaling was carried out with the software XDS (52). Initial phases were obtained using Phaser (53) by the molecular replacement method using the yellow lupine LIPR-10.2B protein (code 3E85) (19) from the Protein Data Bank (54) as a search model. Successive rounds of automatic refinement and manual building were carried out with RefMac5 (55) and Coot (56). The three structural models have been deposited in the Protein Data Bank (57) with codes 4C9C, 4C94, and 4C9I.

## RESULTS

### 

#### 

##### The Fra a Proteins Bind Natural Flavonoids with Different Specificities

**FIGURE 2.**
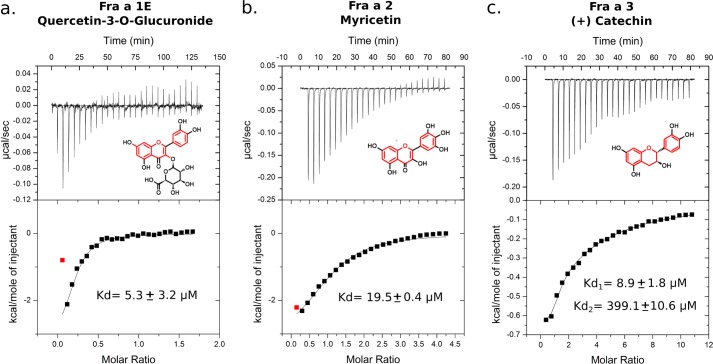
**Fra a proteins bind natural flavonoids.** ITC representative experiments demonstrating binding of different flavonoids to Fra a 1E (*a*), Fra a 2 (*b*), and Fra a 3 (*c*) are shown. Concentrated solutions of quercetin-3-*O*-glucuronide, myricetin, and (+)-catechin (as indicated) were injected into the ITC cell containing the respective protein solutions. The values of the dissociation constants (*K_d_*) are indicated (averaged values over several independent experiments with S.D. between *brackets*). The thermograms for the Fra a 3 (+) catechin pair (*c*) indicate the presence of two distinct ligand binding sites with high (*K_d_*_1_) and low high (*K_d_*_2_) affinities, respectively.

Silencing of *Fra a* genes in strawberry fruits leads to alterations in the accumulation of specific metabolites of the flavonoid pathway, including increased levels of catechin and proanthocyanidins and decreased levels of some anthocyanins and flavonols (28). These compounds are structurally related, as they all contain a flavan nucleus (Fig. 1). We reasoned that Fra a proteins might exert their function through binding to a flavonoid compound containing a flavan nucleus. To test this hypothesis, we performed binding studies with a number of natural compounds from the major branches of the flavonoid biosynthesis pathway (Fig. 1, and see “Experimental Procedures”). ITC experiments allowed the identification of three specific interactions between Fra a proteins and different flavonoid compounds and with affinities in the low micromolar range (Fig. 2). Quercetin-3-*O*-glucuronide binds to Fra a 1E with a dissociation constant (*K_d_*) of 5.3 μm, whereas Fra a 2 binds myricetin with a *K_d_* of 19.5 μm. In both cases, the binding stoichiometry is 1:1. Similarly, ITC experiments demonstrated binding between (+)-catechin and Fra a 3. In this case, the thermograms showed a complex binding curve indicating the presence of two distinct binding sites, which were later confirmed by the crystallographic model (see below). For Fra a 3, thermograms were fitted to a two-site binding model, which indicated a *K_d_* of 8.9 μm and a second site with very low binding affinity (*K_d_* in the higher micromolar range, Fig. 2). ITC experiments failed to detect binding between Fra a 2, the most abundant isoform in the ripe fruit and early precursors in the phenylpropanoid pathway, similar to l-phenylalanine, coumaric acid, cinnamic acid, and caffeic acid. These results demonstrate that Fra a proteins can bind metabolites of the flavonoid pathway with affinities in the low μm range and with different selectivity.

##### The Structure of Apo-Fra a 1E Shows Conformational Flexibility in Loop Regions Surrounding the Ligand Binding Cavity

We attempted to crystallize Fra a 1E, Fra a 2, and Fra a 3 proteins both in the presence and absence of flavonoids. For this purpose, the three Fra a proteins were expressed in *E. coli* and purified to homogeneity (see “Experimental Procedures”). SEC-MALLS experiments showed an apparent *M*_r_ of 18 kDa, 28 kDa, and 17.5 kDa for Fra a 1E, Fra a 2, and Fra a 3, respectively. In the case of Fra a 1E and Fra a 3, these values are in good agreement with the expected molecular masses, indicating that both proteins are monomeric in solution. The estimated molecular weight for Fra a 2 is slightly higher than expected. Fra a 2 did not produce crystals in any of the conditions tested.

The Fra a 1E protein crystallized in two different crystal forms. The two crystal forms belong to the same space group (*P*2_1_2_1_2_1_) but show different unit cell dimensions and molecular arrangement. Crystal form A contains two protein molecules per asymmetric unit, whereas crystal from B contains six independent copies per asymmetric unit. Because Fra a 1E is monomeric in solution, the intersubunit contacts in both crystal forms are likely to be induced by the crystallization process. The first crystals diffracted to 2.2 Å resolution, whereas the second crystal form diffracted to 3.1 Å (see Table 1 for crystallographic data collection and refinement statistics).

**TABLE 1:** **Crystallographic data collection and refinement statistics**

	Fra a 1E (Crystal form A)	Fra a 1E (Crystal form B)	Fra a 3-catechin
**Data Collection**			
Space group	*P*2_1_2_1_2_1_	*P*2_1_2_1_2_1_	*C*222_1_
Unit cell	*a* = 70.02, *b* = 74.42, *c* = 84.04 Å; α = 90°, β = 90°, γ = 90°	*a* = 81.73, *b* = 82.46, *c* = 224.77 Å; α = 90°, β = 90°, γ = 90°	*a* = 137.91, *b* = 206.61, *c* = 174.7 Å; α = 90°, β = 90°, γ = 90°
Resolution	36.03–2.2 (2.32–2.2)*^a^*	30–3.1 (3.27–3.1)*^a^*	30–3.0 (3.16–3.0)*^a^*
No. of observations (overall/unique)	165,127/24,107	104,880/27,085	371,478/49,904
Average redundancy	7.2 (7.3)	3.9 (3.9)	7.4 (7.5)
*R*_p.i.m._	0.034 (0.183)	0.078 (0.307)	0.03 (0.23)
Completeness	99.9 (100%)	96.5 (98)	99.5 (100)
*I*/σ(*I*)	14.0 (4.2)	7.9 (2.6)	21.7 (3.6)

**Refinement**			
Resolution range (Å)	36.03–2.2	29.87–3.1	29.67–3.0
*R*_work_	0.21	0.21	0.18
*R*_free_	0.25	0.26	0.20
No. of non-H atoms			
Protein	2427	7567	6346
Solvent	157	23	12
Ramachandran plot (%)	98.4/1.6/0.0	96.9/3.1/0.0	98.3/5.6/0.1
r.m.s.d.*^b^*			
Bond length	0.019	0.012	0.027
Angles	1.961	1.531	2.692
Average B-factors			
Protein	39.83	58.45	72.45
Ligand			92.94

*^a^* Highest resolution range.

*^b^* r.m.s.d., root mean square deviation.

**FIGURE 3.**
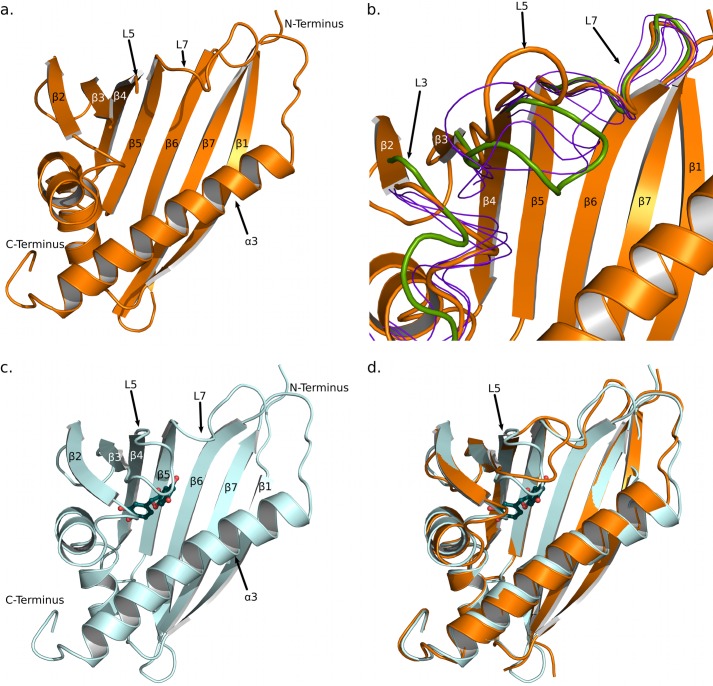
**Structure of the Fra a 1E and Fra a 3-catechin complex.** *a*, Fra a 1E, crystal form A, shown in ribbon representation. *b*, alternative loop conformations in Fra a 1E, crystal form B. The backbone atoms of the six independent molecules in the asymmetric unit have been superposed. Chain F is shown in ribbon representation. The different conformations of the loops L3, L5, and L7 are represented in different colors: *orange*, open conformation (molecule F); *green*, closed conformation (molecule C); and *blue*, intermediate conformations (molecules A, B, D, and E). *c*, Fra a 3-(+)-catechin complex, the protein and ligand are shown in ribbon and ball-and-stick representations, respectively. *d*, superposition of Fra a 1E (*orange*) and Fra a 3-catechin (*cyan*) structures.

Initial phases for crystal form A of Fra a 1E were obtained by the molecular replacement method using the yellow lupine LIPR-10.2B protein (Protein Data Bank code 3E85) (19) as a search model. The final model was refined to a resolution of 2.2 Å with an *R*_work_ and *R*_free_ of 0.21 and 0.25, respectively. The refined model contained two molecules of Fra a 1E in the asymmetric unit. Both molecules showed a similar structure and comprise amino acids 2 to 160. The region corresponding to the β3-β4 loop (amino acids 61–63 and 61–65 in chains A and B, respectively) was disordered and could not be modeled (Fig. 3). The three amino acids from the N-terminal purification tag that remain after TEV cleavage were also visible in the structure. As can be appreciated in Fig. 3*a*, the structure of Fra a 1E conforms to the START fold (2, 9) and consists of a seven-stranded β-sheet, two short helical segments between strands 5 and 7, and a long α-helix at the C terminus. The β-sheet adopts a slightly curved shape with the long C-terminal α-helix (α3) juxtaposed against the concave side, which creates a cavity in the middle of the protein. Two short helical segments (α1 and α2) close the cavity on one of its sides, whereas the other extremity is open and accessible to the solvent. The open end of the cavity was surrounded by loops α2-β2, β3-β4, β5-β6, and β7-α3, designated here, respectively, as L3 (amino acids 35–39), L5 (amino acids 60–65), L7 (amino acids 89–96), and L9 (amino acids 125–130), and by the N-terminal half of the last α-helix (α3). A number of well ordered water molecules were found inside the cavity. The overall configuration of the Fra a 1E backbone was similar to those of other START and PR-10 proteins, like Bet v 1 from *Betula berrucosa* (3, 5) or LIPR-102.B from yellow lupin (19). The major differences were found in the conformation of the loops surrounding the open end of the cavity.

Initial phases for Fra a 1E crystal form B were obtained by the molecular replacement method, using the refined model of crystal form A. The final model was refined to a resolution of 3.1 Å with an *R*_work_ and *R*_free_ of 0.21 and 0.26. The refined model contains six molecules of Fra a 1E in the asymmetric unit. None of these molecules contained catechin inside the cavity. However, a molecule of catechin was found at the surface of molecule F between two Fra a 1E subunits in adjacent asymmetric units. The nature of the interaction suggests that this catechin molecule has been captured as a consequence of the crystallization process. As expected, the six molecules in crystal form B show a very similar structure, which is also similar to that of crystal form A (see Fig. 3*b*). However, a high degree of conformational variation is observed in the loop regions L3, L5, and L7. In particular loop L5, shows a distinct conformation in every of the six molecules found in the asymmetric unit. These conformations range from a “closed” conformation, represented by molecule C, in which loop L5 is folded over the central cavity of the protein and approaches the C-terminal α-helix (colored *green* in Fig. 3*b*), to an “open” conformation represented by molecule F, in which loop L5 is at a maximal distance from the C-terminal α-helix (colored *orange* in Fig. 3*b*). When these two molecules are superimposed, the positions of the Cα atoms of serine 63, in the middle of loop L5 are >9 Å apart. This indicates that loops L3, L5, and L7 surrounding the central cavity of Fra a show considerable conformational flexibility and that loop L5 is capable of adopting both open and closed conformations.

##### Structure of the Fra a 3 Protein in Complex with Catechin

In the case of Fra a 3, crystals were obtained only in the presence of catechin, a natural flavonoid present in strawberries. The structure of the Fra a 1E protein was used to obtain initial phases by the molecular replacement method. The final Fra a 3 model was refined to a resolution of 3.0 Å with an *R*_work_ and *R*_free_ of 0.18 and 0.20, respectively (see Table 1 for crystallographic and refinement statistics). The crystallographic model contains five protein chains and six molecules of catechin. The five protein chains in the asymmetric unit show a similar configuration. The 159 amino acids of the Fra a 3 sequence could be modeled in all the chains except for amino acids 61 to 65 (corresponding to loop L5) of chain D that were not modeled due to weak density. SEC-MALLS analysis indicated that Fra a 3 is a monomer in solution. Hence, the intersubunit interactions between Fra a 3 subunits are likely to be induced by the crystallization process. For simplicity, we will refer to chain A for the rest of the text.

**FIGURE 4.**
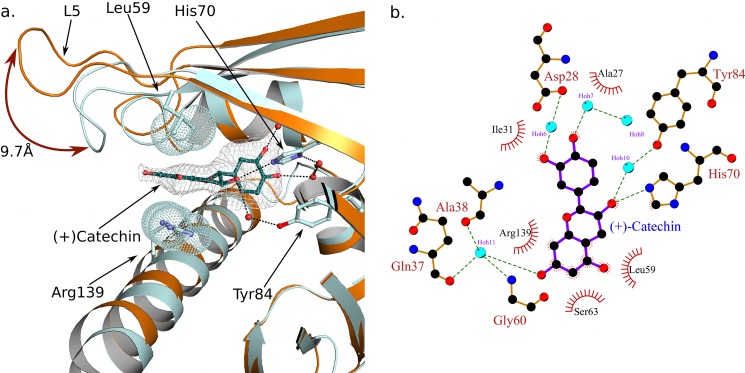
**Binding of (+)-catechin to the Fra a 3 cavity involves both polar and hydrophobic interactions and a closed conformation of loop L5.** a. Structural superposition of Fra a 1E (*orange*) and Fra a 3 (cyan) around the loop 60–64. Protein chains are shown in ribbon representation. The catechin molecule and the side chains of the binding residues 59, 70, 84, and 139 are shown in ball and stick representation. Electron density around catechin is shown (omit map). The Van der Waals spheres of the distal atoms of the side chains of residues 59 and 139 are indicated by *dot* representation. The closed conformation of Fra a 3, stabilized by interactions with the ligand, is shown. *b*, Ligplot (71) representation of the molecular interactions between (+)-catechin and Fra a 3. *Green dashed lines* indicate hydrogen bonds. Hydrophobic interactions are indicated by *red semicircles*.

As expected, Fra a 3 shows a typical START fold (Fig. 3*c*) and high structural similarity with Fra a 1E (both structures can be superimposed with an RSMD of 0.82 Å over 148 Cα atoms). The major differences between Fra a 3 and Fra a 1E are found in the loops surrounding the entry to the cavity (Fig. 3*d*, see below). All of the molecules of Fra a 3 in the asymmetric unit showed additional density inside the cavity. This density could be easily interpreted as (+)-catechin (Fig. 4*a*). The sixth (+)-catechin molecule in the Fra a 3 crystals is not found inside the cavity but at a surface location at the interface between three Fra a 3 proteins in adjacent asymmetric units. The presence of this additional molecule is in agreement with the ITC results that identified the presence of a low affinity binding site. In the crystal structure, this second catechin molecule is stabilized by contacts to three different protein molecules; however, Fra a 3 is monomeric in solution. Hence, this second site is unlikely to play a major physiological role.

The catechin molecule inside of the Fra a 3 binding cavity is stabilized both by polar and hydrophobic interactions (Fig. 4*a*) and shows the same conformation in the five copies within the asymmetric unit. The flavan nucleus is oriented with its long axis approximately parallel to the helix α3 and with the B ring pointing toward the bottom of the cavity. Interestingly, as in the case of the structurally related PYR/PYL/RCAR abscisic acid receptor proteins (15–18), many of the polar interactions are mediated by water molecules (Fig. 4*b*). The two hydroxyl groups in the C ring are involved in a hydrogen bond network with water molecules and residues Asp-28, and His-70. Similarly, the hydroxyl group in position 6 of the A ring, at the opposite end of the catechin molecule, is stabilized by a series of hydrogen bond interactions with a water molecule, the hydroxyl group of Ser-63 and backbone atoms in residues Gln-37, Ala-38, and Gly-60. The hydroxyl group in position 3 of the central ring of catechin makes hydrogen bond interactions with His-70 and with a water molecule, which in turn is hydrogen bonded to Tyr-84. The aromatic A ring is pinned between Leu-59 and the guanidinium group of Arg-139. Finally, the non-polar groups of catechin are surrounded by hydrophobic side chains, including Ile-31, Val-39, Leu-59, and Leu-143. These interactions stabilize the catechin molecule in the Fra a 3 cavity and restrict the rotation around the single bond linking rings C and B, whose central planes are oriented at an angle of ∼90°.

##### Binding of Catechin to Fra a 3 Involves a Closed Conformation of Loop L5

The most notable difference between the Fra a 1E and Fra a 3 structures presented here are the presence of the ligand and the conformation of the loop L5, at the edge of the binding cavity (see Fig. 3). In the Fra a 3 structure, the loop L5 wraps around one end of the catechin molecule and approaches the C-terminal α-helix (α3) on the opposite side restricting the access to the cavity. At the same time, helix α3 of Fra a 3 shows a slight bend at its center toward loop L5, whereas loop L3 approaches loop L5 and the ligand (see Fig. 3*d*). This closed conformation is stabilized by interactions with the catechin molecule. Namely, polar interactions between the hydroxyl in position 6 of the A ring of catechin, Ser-63 in loop L5 and backbone atoms in residues Gln-37, Ala-38 of loop L3, and the hydrophobic interaction with Leu-59 in loop L5 described above. Finally, the bending of helix α3 contributes to the packing of the guanidinium group of Arg-139 against the A ring of catechin. This conformation produces a very compact structure with the catechin molecule enclosed inside the cavity. The cavity also shows a significant reduction in volume as compared with the Fra a 1E structures (1646.4 A^3^ for Fra a 3, 2204.8 A^3^ for molecule A in the Fra a 1E crystal form A). Molecule C in crystal form B of Fra a 1E, is the one that more closely resembles the Fra a 3 catechin complex with loop L5 approaching helix α3 (Fig. 2*B*). However, in this case, loop L3 adopts an open conformation, and the bend in helix α3 is not present. This suggests that loops L3, L5, and L7 of Fra a proteins may display a high level of conformational flexibility, but the presence of a ligand would be necessary to promote coordination between critical secondary structural elements leading to a fully closed conformation.

## DISCUSSION

Fra a proteins are members of the pathogenesis-related 10 family and are required for the normal accumulation of flavonoids and the development of color in strawberry fruits (26, 28). However, their function, as that of PR-10 proteins in general, is still not clearly understood. The data presented here demonstrates that Fra a proteins bind natural flavonoids, providing for the first time mechanistic insight on the function of these proteins in the control of flavonoid biosynthesis.

**FIGURE 5.**
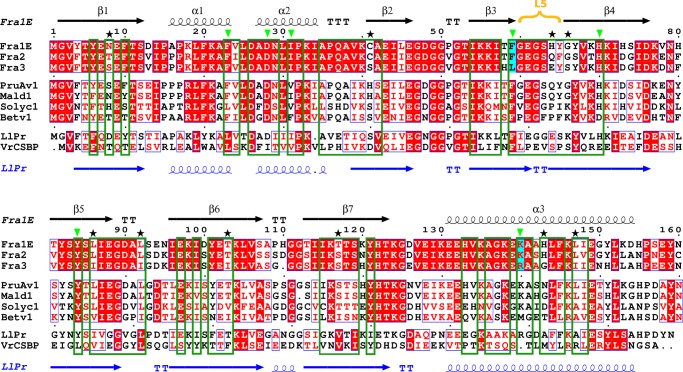
**Multiple sequence alignment of the strawberry Fra a 1E, Fra a 2, and Fra a 3 proteins and other related Bet v 1/START proteins.** The secondary structural elements correspond to the Fra a 1E (*black*) and LIPR-10.2B (*blue*) structures. The L5 loop has been highlighted in *yellow*. Residues oriented toward the cavity and residues involved in catechin binding (for Fra a 3) are indicated by *dark green boxes* and *light green triangles*, respectively. Positions showing sequence variations, which are either important for catechin binding (*cyan*) or facing the cavity (*black stars*), are also indicated. The sequence alignment was performed using ClustalW (EBI server), and the figure was generated by ESPript (72).

ITC experiments show that Fra a proteins can bind metabolites of the flavonoid pathway with affinities in the low μm range and with different selectivity. The three ligands identified in this study have been shown to be present in fruits as well as other parts of the strawberry plant (45, 49, 50, 58) and accumulate at the same organ and developmental stage where the highest expression levels of the Fra a proteins occur (27). The structure of the Fra a 3-catechin complex provides details on the mechanism of ligand binding and stabilization. The catechin molecule adopts a linear disposition with its long axis approximately parallel to the axis of the long C-terminal α-helix of Fra a 3. It is stabilized by polar interactions with the side chains of Asp-28, Ser-63, His-70, Tyr-84, and Arg-139 and backbone atoms of residues Gln-37, Ala-38, and Gly-60, whereas the non-polar groups of catechin are surrounded by hydrophobic side chains including Ile-31, Val-39, Leu-59, and Leu-143. The amino acids facing the cavity in the three Fra a proteins are generally conserved. However, variability is observed between the three proteins at certain positions (Fig. 5), which could explain their different selectivity toward ligands. For example key amino acids Leu-59 and Arg-139, involved in Fra a 3 (+)-catechin interaction are replaced by Phe and Lys in both Fra a 1E and Fra a 2.

**FIGURE 6.**
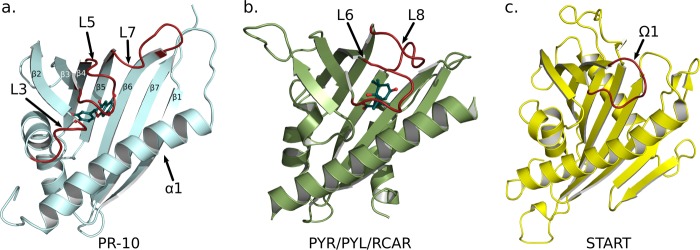
**Function of PR-10, PYR/PYL/RCAR, and START proteins involve conformational changes in loop regions.** *a*, Fra a 3 in ribbon representation with bound (+)-catechin. The flexible loops L3, L5, and L7 are shown in *red. b*, the abscisic acid receptor PYR1 (Protein Data Bank code 3K90) in ribbon representation. The gating loops undergoing conformational changes hormone binding and receptor activation are depicted in *red. c*, the START domain of the CERT protein (Protein Data Bank code 3H3S) in ribbon representation. The Ω1 loop is shown in *red*.

Comparison of the structures of the apo forms of Fra a 1E and the Fra a 3-catechin complex indicates that Fra a proteins show considerably flexibility in the loop regions surrounding the cavity (loops L3, L5, and L7) and that ligand-binding induces important conformational changes. Fra a 3 adopts a more compact structure with a closed conformation of loop L5 that traps the catechin molecule inside the cavity. Interestingly, loop L5 in Fra a proteins is structurally equivalent to the Ω1 loop of the mammalian START proteins (59) and the β3-β4 loop of the plant PYRL/PYL/RCAR hormone receptors (18), which also adopt closed conformations upon ligand binding (see Fig. 6). In the case of the mammalian START proteins, these conformational changes are thought to play a role in lipid extraction and solubilization (59–61), whereas in the plant abscisic acid receptors the closed conformation stabilizes the hormone inside the cavity and promotes interaction between the receptor and protein phosphatases of the class 2C, leading to the activation of the ABA signaling pathway (14–16, 18). This suggests that ligand-induced conformational changes are a conserved feature in the START protein superfamily and might also play an important role in the function of other members of the PR-10 family.

The structural analysis presented here together with the molecular mechanisms previously described for LPTs and PYR/PYL/RCAR proteins suggest two possible functions for the Fra a proteins at molecular level. Fra a proteins could act as transporters or “chemical chaperones” binding to flavonoid intermediates and making them available to processing enzymes. The key enzymes of the phenylpropanoid and flavonoid biosynthesis pathways, including PAL, chalcone synthase, and C4H have been shown to co-localize to the endoplasmic reticulum in different plant species. These enzymes form multi-protein complexes at the cytosolic side of the membrane where synthesis of many flavonoid and phenylpropanoid compounds occurs (44, 62, 63). This association in multiprotein complexes has been proposed to help sequester unstable or toxic intermediates and to control the metabolic flux among the multiple branches of the pathway, thereby determining which compounds are synthesized preferentially (63). Fra a proteins might form part of these complexes, contributing to limit diffusion of intermediates and making them available to downstream processing enzymes. Fra a proteins could also be involved in the transport of flavonoids from the ER to other cellular membranes, such as the tonoplast or the plasma membrane. Indeed, anthocyanins and other conjugated flavonoids such as glycosylated catechin and epicatechins are translocated to and accumulated into the vacuole through the action of specific membrane transport proteins, whereas other flavonoid compounds are secreted to the apoplast through the plasma membrane, especially in roots (44, 64–66). In this case, Fra a proteins might have a function analogous to that of the mammalian START proteins that act as cytosolic transporters of lipids shuttling between different cellular membranes (11–12).

Another possibility is that Fra a proteins might play a role as regulatory components involved in intracellular signaling. In maize, *Arabidopsis* and other species, the genes coding for enzymes involved in phenylpropanoid and flavonoid biosynthesis are regulated at a transcriptional level through the activity of MYB and bHLH type transcription factors (32, 62, 67). Moreover, it has been recently shown that a blockage in downstream flavonoid processing enzymes results in transcriptional inhibition of PAL and that this inhibition is dependent on the accumulation of flavonoids, demonstrating that the expression of structural genes is mediated by a metabolic intermediate downstream of naringenin (68). The capacity of Fra a proteins to bind specific flavonoids suggests that they could play a role as signaling components, monitoring the metabolic flux through different branches of the pathway and influencing the expression level of specific regulatory genes. This would be consistent with the effect of Fra a silencing on the transcriptional activity of PAL and chalcone synthase genes and the altered accumulation of certain flavonoids (28).

Close homologues of the Fra a proteins have been found in apple, peach, and tomato, some of which are also expressed to high levels during fruit ripening (69–70). The amino acids involved in the Fra a 3-catechin interaction are also highly conserved in these proteins (see Fig. 5), which suggests that these proteins might also have the capacity to bind structurally close flavonoids. However, other PR-10 proteins show more divergent amino acids sequences in the cavity region (see Fig. 5) and might bind other ligands. The phenylpropanoid and flavonoid biosynthesis pathway is responsible for the production of a large proportion of secondary metabolites in plants and shows a high degree of variability among species. It is not only involved in development of color in fruits and flowers, but it is also important for many other biological functions in plants, including defense against pathogens, insect attraction, and pollination and UV protection, among others. The structural analysis of the Fra a proteins suggests that PR-10 proteins, which are widespread in plants, might function in the control of flavonoid or other secondary metabolic pathways in plants.

## Footnotes

5 The abbreviations used are:

PR-10: pathogenesis-related 10
PAL: phenylalanine ammonia-lyase
ITC: isothermal titration calorimetry
START: StAR-related lipid transfer
PYR: pyrabactin resistance
PYL: pyrabactin resistance-like
RCAR: regulatory components of ABA receptors
TEV: tobaco etch virus.
